# Dental trauma characteristics in the primary dentition in Greifswald, Germany: a comparison before and after German unification

**DOI:** 10.1007/s40368-021-00606-5

**Published:** 2021-02-13

**Authors:** M. A. Eissa, M. Mustafa Ali, C. H. Splieth

**Affiliations:** 1 Department of Preventive and Paediatric Dentistry, University of Greifswald, Walther-Rathenau-Straße 42, 17475 Greifswald, Germany; 2 Taibah University College of Dentistry, Jannadah Bin Ummayah Road, Medinah, Saudi Arabia

**Keywords:** Trauma, Primary teeth, Deciduous teeth, Tooth injuries, Germany

## Abstract

**Background:**

The data on the distribution and pattern of dental trauma in the primary dentition are very rare.

**Aim:**

To investigate primary dental trauma patterns in relation to gender, age, time and cause for a recent paediatric cohort and to compare this with a historic one before German unification.

**Methods:**

This retrospective study compared the patterns of dental trauma injuries at the trauma centre at Greifswald University/Germany for a recent paediatric cohort (2014–16, 103 children) with a historic one (1974–1989, *n* = 120). The data contained details on etiology, injury types and treatment delivered for 450 injured teeth (247 historic, 203 recent).

**Results:**

In both cohorts, the occurrence was more common in males than females (63%/55%, resp.) with an age peak from 2 to 6 years. Maxillary incisors were most affected (89.6%/88.6%, resp.) and periodontal ligament injuries dominated (77.8%/90.3%, resp.).

Almost half of the injuries occurred at home (46.6%), mostly due to falling (48.5%) or during playing (37.8%) in the recent paediatric cohort which provided better forensic data due to insurance issues and potential concern about child abuse. Advice and follow up was the most common approach in the recent paediatric cohort (76%).

**Conclusion:**

Even after 30 years and a change in the health care system due to German unification, the patterns of dental traumatic injuries in the primary dentition were similar.

## Introduction

The epidemiologic data on dental trauma injuries in the primary dentition are very rare and often contradictory. Bastone et al. (2000) criticized the use of different trauma classifications used and found variations for the study locations and included age groups possibly leading to a bias in the reported outcome. According to Cardoso and De Carvalho Rocha (2002), the greatest incidence of primary tooth trauma occurs during motor coordination development at the age of 2–3 years. In addition, a bias for the prevalence of dental trauma is very likely since not all cases report to the dental clinics or recorded uniformly. For example, Arikan et al. (2010) reported that parents do not tend to seek medical care in the absence of acute symptoms.

Trauma to the primary dentition may result in fracture of the tooth, the alveolar bone, luxation or subluxation injuries, concussion injuries as well as avulsion of the tooth. Periodontal tissue injuries were reported to be the most common type of injuries for trauma in primary teeth (Bastone et al. 2000; Andreasen 1970; Skaare and Jacobsen 2005). Bagattoni et al. (2017) reported avulsion injuries to be the commonly associated injuries with primary tooth trauma in children with special health care needs. Discoloration of the injured primary tooth is a common consequence which may be either persistent or temporary, characterized by a direct relationship between tooth discoloration and pulp necrosis in traumatized deciduous teeth (Boorum and Andreasen 1998; Cardoso and De Carvalho Rocha 2010).

Not only does dental trauma affect the primary dentition, but also its effect extends sometimes to the permanent successors, such as esthetically relevant discoloration and hypoplasia (Sennhenn-Kirchner and Jacobs 2006). Furthermore, developmental effects, such as malformation of the permanent successor, impaction and eruption disturbances could result too from such a trauma due to the close relationship between the injured primary tooth apex and the developing permanent tooth germ (Andreasen and Ravn 1971; Diab and Elbadrawy 2000).

The International Academy of Dental Traumatology (IADT) has issued a set of guidelines based on the literature for clinical management of tooth trauma including the evaluation on the history of the injury and its possible association to child abuse, especially in children younger than 5 years old (Kellogg 2005; Becker et al. 1978). Malecz (1979) noted that 25 cases of suspected child abused children in 1979 were reported by pediatric dentists. Moreover, Becker et al. (1978) reported 65% of 260 child abused children had suffered from orofacial injuries. This highlights the role of the paediatric dentist in discovering, documenting and reporting the presence of a possible child abuse.

Trauma evaluation requires a radiographic examination which is necessary to highly recommended to assess the extent of injury regarding the supporting tissues, the stage of root development as well as the relationship of the injured tooth to the permanent successor, as vitality and percussion tests are not reliable in small children (Malmgren et al. 2012). Owing to these diagnostic and often also cooperation problems, extraction is usually the treatment of choice (Malmgren et al. 2012; Needleman 2011).

As traumatic dental injuries involve very high costs on patients and insurance companies and complicated injuries can have negative effects on the quality of life of preschool children, investigating the causes and patterns of trauma in primary teeth are highly needed (Borum and Andreasen 2001; Aldrigui et al. 2011).

The city of Greifswald, where this study occurred, lies in North East Germany, and was part of the communist eastern state before the German unification, where the health insurance system was under the authority of the socialist party and its ideology (Hurst 1991). The data on the progress of primary tooth trauma patterns, aetiology and management in these two completely different health systems is missing in the literature. Thereupon this study aims to investigate the distribution patterns of dental trauma in relation to gender, age, cause and management in a recent paediatric cohort and to compare findings with a historic paediatric cohort before the German unification.

## Materials and methods

This retrospective study compared the prevalence of primary tooth trauma, its causes and consequences in a recent paediatric cohort with a historic one before the German unification. The Ethics Committee at the Greifswald University approved the study on the 15th of March 2016 (Ethics approval Nr. BB 028/16). The data for the recent paediatric cohort were collected from the 2014 to 2016 records of the Department of Pediatric Dentistry in Greifswald University being the current first entry gate for dental trauma. The data regarding the historic paediatric cohort during the socialistic, East German area were gathered from the records of the Department of Oral and Maxillofacial Surgery which was the primary entry point to the University Dental Clinics then (1974 and 1989).

The parameters included: age, gender of the traumatized child, cause and place of the trauma and the type of the trauma with the treatment offered.

The recent paediatric cohort data were classified according to the IADT guidelines (Malmgren et al. 2012). Whereas in the historic paediatric cohort the trauma was recorded in categories from 1 to 5 for periodontal ligament injuries: grade 1 represented the occurrence of subluxations, grade 2 lateral luxation, 3 intrusions, 4 extrusions and 5 avulsion. Hard tissue injuries were graded from1 to 4: grade 1 for enamel fracture, 2 enamel dentine fracture without pulp involvement, 3 enamel dentine fracture with pulp involvement, 4 crown–root fractures. Still, this grading could easily be converted to the IADT system. Combined injuries were registered when more than one symptom was mentioned. The data were entered in an Excel spreadsheet, transferred and then statistically analyzed using SPSS version 20. Descriptive statistics were calculated for all the collected data. We used the Chi-square test, the independent samples *t* test and Wilcoxon signed rank test to assess the differences between the two paediatric cohorts.

## Results

A total number of 223 patients was included in this retrospective study with 103 patients in the recent pediatric cohort and 120 patients in the historic one. The gender distribution revealed that more males than females reported with trauma to the dental clinics: 63% versus 37% in the recent paediatric cohort and 55% versus 45% in the historic one (Table 1).

**Table 1:** Demographic description of the sample

	Total cohort *N* = 223	Recent cohort *N* = 103	Historic cohort *N* = 120
Gender (patient level)			
Male *n* (%)	131 (58.8%)	65 (63.1%)	66 (55%)
Female *n* (%)	92 (41.2%)	38 (36.9%)	54 (45%)
Age mean in years (± SD)	3.85 (± 2.05)	3.89 (± 2.27)	3.84 (± 1.85)
Number of teeth affected			
One (%)	84 (38%)	45 (44%)	39 (32%)
Two (%)	90 (40%)	39 (38%)	51 (42%)
More than 2 teeth (%)	49 (22%)	19 (18%)	30 (25%)
Mean number of affected teeth	1.9	1.97	2.05
Total number of teeth	450	203	247
Gender (tooth level)			
Male *n* (%)	265 (59%)	139 (68%)	126 (51%)
Female *n* (%)	187 (41%)	64 (32%)	123 (49%)

**Fig. 1:**
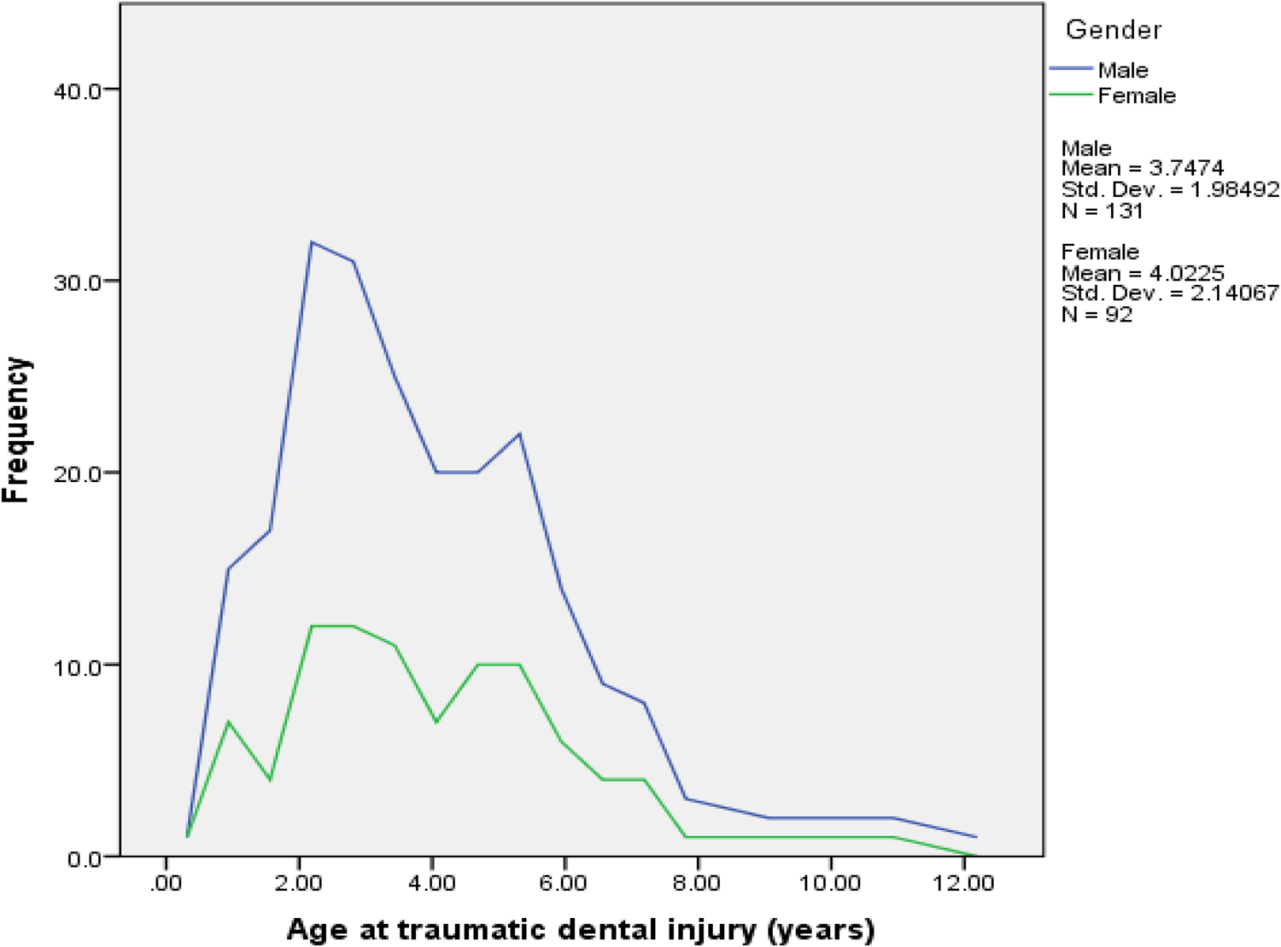
Age distribution of children reporting to a specialist centre due to dental traumatic injuries in the primary dentition

The mean age of the patients is 3.8 years old in both samples (Table 1), with a range from 0 to 12 years old. The peak of trauma was between 2 and 6 years old (Fig. 1) The total number of teeth injured was 450 deciduous teeth: 203 in the recent paediatric cohort and 247 in the historic one.

### Location and causes of traumatic dental injuries

In the recent paediatric cohort, most of the traumatic primary tooth injuries occurred at home (47%, *n* = 48) followed by kindergartens (33%, *n* = 34) mainly due to falling and slipping (49%) or during playing (38%, Table 2). Due to insurance issues and the concern on child abuse, the forensic documentation in the recent paediatric cohort was far more detailed than in the historic pediatric cohort.

**Table 2:** Distribution of the causes of primary tooth trauma among children

	Recent *n* = 103 (%)^a^	Historic *n* = 120 (%)^a^
Falling and slipping	50 (48.5%)	8 (6.6%)
Playing accidents	39 (37.8%)	18 (15%)
Others	11 (10.6%)	7 (5.8%)
Missing	3 (2.9%)	87 (72.5%)

^a^On a patient level

### Patterns of traumatic dental injuries

**Fig. 2:**
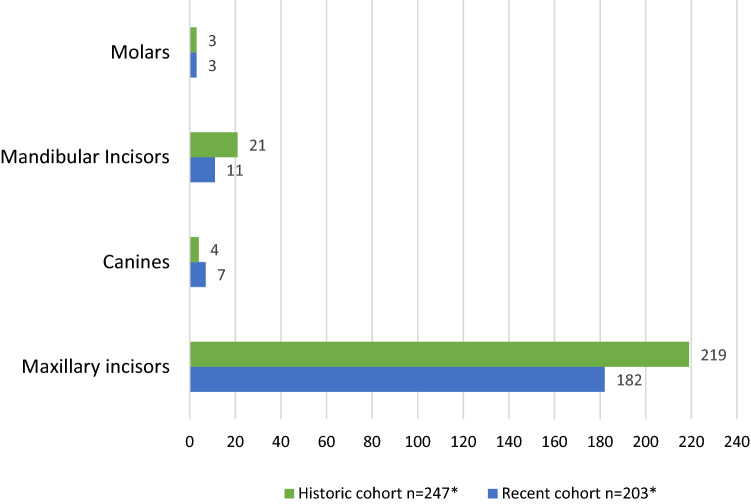
The distribution of the traumatized primary teeth in the recent and historic cohorts

The distribution of the injuries showed that the most affected teeth in the recent and historic paediatric cohorts were the maxillary incisors, while the least affected teeth were the molars (Fig. 2).

Traumatic injuries were classified into hard tissue injuries, periodontal tissue injuries, soft tissue injuries and combined injuries. PDL injuries were the most common type of injuries in the recent and historic paediatric cohorts (Table 3). Hard tissue injuries include all the traumas affecting the dental structure or the alveolar bone, including enamel chipping, fracture of the teeth with pulp exposures or without, root and alveolar fractures.

**Table 3:** Distribution of the type of injuries resulting from primary tooth trauma

	Hard tissue injuries^b^	PDL injuries^b^	Soft tissue injuries^a^	Combination injuries^b^	Missing^b^
Recent cohort *n* = 203^b^	47 (23.2%)	158 (77.8)	57 (55%)	8 (3.9%)	3 (1.4%)
Historic cohort *n* = 247^b^	37 (14.9%)	223 (90.3%)	31 (25.8%)	17 (6.9%)	4 (1.6%)

^a^On a patient level [*N* (recent) = 103, *N* (Historic) = 120]

^b^On a tooth level

Twenty-three percent of the children in the recent paediatric cohort presented with fracture injuries (*n* = 47), most of them were just uncomplicated enamel dentine fracture with no pulp exposure, followed by complicated enamel dentine fracture with pulp exposure. However, a coronal fracture involving only the enamel was the most common type of fracture injury recorded in the historic paediatric cohort (*p* value > 0.05). While the least common in both cohorts was crown root fractures with pulp exposure (Table 4).

**Table 4:** Distribution of the types of hard tissue injuries in both cohorts

	Recent cohort *n* = 203 (%)^a^	Historic cohort *n* = 247 (%)^a^
Enamel fracture	2 (0.9%)	18 (7.2%)
Enamel–dentin fracture without pulp exposure	22 (10.8%)	10 (4%)
Enamel–dentin fracture with pulp exposure	13 (6%)	4 (1.6%)
Crown–root fracture with pulp exposure	2 (0.9%)	0
Root fracture	4 (1.8%)	0
Alveolar fracture	4 (1.8%)	5 (2%)

^a^On a tooth level

**Fig. 3:**
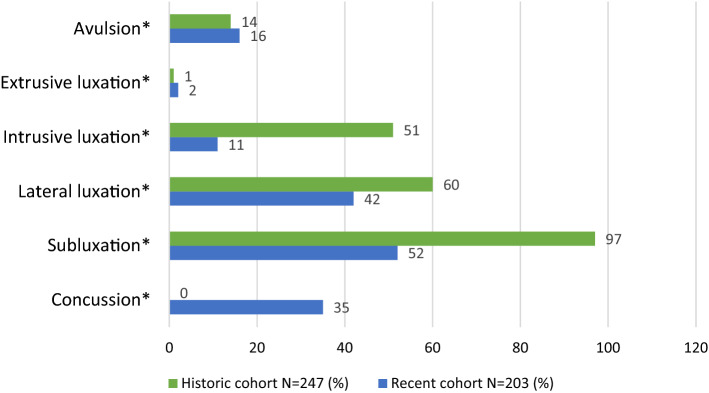
Distribution of PDL injuries in the recent and historic cohorts

Injuries of the periodontal ligament (PDL) were defined as all the traumatic injuries affecting the tooth periodontium in any form ranging from concussions to complete avulsion of the teeth (luxation injuries), about 80% of the whole sample (*n* = 223 patient) suffered from PDL injuries. Although subluxation was the most common PDL injury in both cohorts, extrusive luxation was the least common. No concussion injuries were reported at all in the historic paediatric cohort (Fig. 3).

Soft tissue injuries which present with a wide range of symptoms such as lip injuries (swelling, abrasions and hematomas), gingival injuries, tongue injuries, intraoral mucosal as well as extraoral injuries affecting cheek, chin or other facial aspects are not the primary domain of the dental profession, but they are also recorded and possibly also treated. Lip injuries were the only relevant recorded soft tissue injury in both cohorts (Table 5).

**Table 5:** Distribution of soft tissue injuries in the recent and historic cohort

	Recent cohort *N* = 103 (%)^a^	Historic cohort *N* = 120 (%)^a^
Lip injuries	36 (35%)	26 (21.7%)
Gingival injuries	15 (14.6%)	2 (1.7%)
Extraoral injuries	5 (4.9%)	1 (0.8%)
Tongue injuries	2 (1.9%)	2 (1.7%)
Intraoral soft mucosal injuries	4 (3.9%)	1 (0.8%)
Combined injuries	9 (8.7%)	1 (0.8%)
No soft tissue injury reported	46 (44.7%)	89 (74.2%)

^a^On a patient level

The intervention method used to manage PDL injuries differed from advice to extractions, splinting, suturing, trepanation or even referral to a surgeon. In the recent paediatric cohort, advice and follow up was the most common intervention approach (*n* = 111, 76%), while in the historic paediatric cohort extractions were more frequent (*n* = 48, 23%), although the difference was not statistically significant (*p* value > 0.05). Unfortunately, the provided intervention method was missing in 65% of the historic paediatric cohort records on PDL injuries interventions (Table 6).

**Table 6:** The distribution of the intervention methods used to manage PDL injuries in the recent and historic pediatric cohorts

*n* = 352^a^ PDL injured teeth	Recent cohort *n* = 146^a^	Historic cohort *n* = 206^a^
Advice	111 (76%)	17 (8.3%)
Extractions	16 (11%)	48 (23.3%)
Splinting	2 (1.4%)	6 (2.9%)
Referral to a surgeon	4 (2.7%)	0
Suturing	10 (6.8%)	0
Trepanation	0 (0%)	1 (0.5%)
Missing data	2 (1.4%)	134 (65%)

^a^On a tooth level

Moreover, hard tissue injuries in the recent sample were most commonly handled by reassurance and advice as well (*n* = 23, 53%), followed by immediate extractions (*n* = 13, 30%). The historic paediatric cohort lacked information on the treatment of hard tissue injuries in 50% of the cases (Table 7).

**Table 7:** The distribution of the intervention methods used to manage hard tissue injuries in both cohorts

*N* = 63^a^	Recent cohort *n* = 43^a^	Historic cohort *n* = 20^a^
Advice	23	5
Restorations	6	1
Extractions	13	4
Referral to surgeon	1	0
Missing data	0	10

^a^On a tooth level

## Discussion

East Germany has experienced a severe structural change in almost any aspect of daily life, including the structure of the health care system, after German unification due to the conversion from a socialist system to a Western market economy. Therefore, it is amazing that the patterns of dental trauma in the primary dentition treated at a specialist centre, such as the university clinics remain very similar over more than thirty years and despite immense societal changes.

The sample size of the present study was representative for the region and comparable to other international and German trauma-related studies (Skaare and Jacobsen 2005; Blockland et al. 2016). Still, most of these studies carry the risk of a selection bias, as patients treated in private dental offices or in the hospital are not included. Furthermore, some parents might not attend a dentist for minor dental trauma injuries (Odersjö et al. 2018). Thus, conclusions can be made only on the range of injuries and the according treatment presenting at a specialist centre and not on the fully epidemiology of dental trauma. However, this study contains valid information on how to train dental specialists for the management of dental trauma injuries or where to aim for preventive measures. The patterns and distributions of dental trauma in the primary dentition seem to be quite universal—even over time—with an age peak of small children of 2–4 years, predominantly more boys being affected while playing or just moving and mostly one injured upper central incisor (Skaare and Jacobsen 2005; Blockland et al. 2016; Beltrão et al. 2007).

Luxation injuries which affect the periodontal ligament were the most common injury in primary teeth, in both paediatric cohorts in this and other studies (Andreasen and Ravn 1972; Glendor et al. 1996). This is attributed to the shorter root and decreased attachment of primary teeth and the high elasticity of the PDL ligaments and surrounding bone. Thus, training general dentists and specialists to manage luxation injuries in the primary dentition of small children would be beneficial. Dental hard tissue fractures were recorded less frequently which coincides also with other studies (Sandalli et al. 2005; Mahmoodi et al. 2015).

Owing to the young age of the children, often very little cooperation for complex treatments, predominately luxation injuries and no space maintaining function of primary incisors, treatment was usually restricted to advice and monitoring or in the fewer, more severe cases to extractions as in other studies (Sandalli et al. 2005; Mahmoodi et al. 2015). The observed pattern of dental trauma injuries and the according treatment followed the recommendations and guidelines of the IADT (Malmgren et al. 2012). Only in the historical paediatric cohort a higher proportion of about one third of the cases were treated with immediate extractions which possibly could be attributed to the primary examination at the Department of Oral and Maxillofacial Surgery at that time.

The clear increase in documentation regarding the location and cause of trauma over time reflects the change in the health care system and the accompanying greater relevance of costs and insurance issues as well as the increased awareness of potential child abuse.

In general, this study shows that the pattern of traumatic dental injuries in the primary dentition presenting at a specialized care facility has not changed significantly even after more than 30 years and despite the enormous structural changes in the political, social and health care systems. The main difference was that the documentation in the historic sample was less detailed, especially regarding forensic issues which are of greater concern nowadays (Charangowda 2010). Only precise documentation of all clinical aspects particularly in suspected cases of child abuse will fulfill the requirements of legal proceedings (Cairns et al. 2005).

Andreasen and Kahler (2015) also noted that inaccurate and incomplete data could result from the absence of a standardized documentation and examination protocol which would be necessary to allow for more valid scientific evaluation to improve prevention and treatment of dental trauma (Sae-Lim et al. 1995). This information should be collected in standardized national or international documentation databases to analyse parameters regarding dental trauma injuries in the primary dentition more systematically.

## Conclusion

The pattern of dental trauma in the primary dentition has not changed much in east Germany even with two different political, social and medical systems and a time lap of 30 years.

- Luxation injuries in rather small children dominated.
- Current documentation and treatment followed basically the recommendations of IADT.
- Injury patterns of dental trauma in the primary dentition seem to be rather universal due to the activities of small children and their anatomic conditions. Trauma to primary dentition occurred mostly at home or in nurseries being hard to avoid due to falls or accidents during normal playing.
- Clear improvements could be observed in the precision of documentation and partially in the classification of dental trauma for the more recent paediatric cohort.
